# Bibliographical Notices

**Published:** 1854-01

**Authors:** 


### EDITORIAL DEPARTMENT.

### BIBLIOGRAPHICAL NOTICES.

*The Use and Abuse of Alcoholic Liquors in Health and Disease.* By WILLIAM B. CARPENTER, M. D., F. R. S., &c. Philadelphia. Blanchard & Lea, 1853.

THIS little book, as is very well known, is the treatise which took the prize of one hundred guineas offered for the best essay on the use of alcoholic liquors in health and disease. The great eminence of the author as a physiologist, and the unanimity with which the distinguished medical gentlemen, selected as a committee of judges, awarded the prize, bespeak for his opinions the most respectful consideration, and render it a matter of no little temerity to dissent from his conclusions.

These conclusions are, briefly, that it is safe to argue from the phenomena of a poisonous over-dose of alcohol, to those of the habitual *excessive* indulgence in it, especially as the latter have been determined by experience to be precisely what we might expect from a careful study of the former: that the moderate use of these beverages undoubtedly predisposes to disease; that they cannot increase the ability of a man to undergo bodily or mental labor; that they are generally insufficient to counteract a deficiency of power in the system to carry on the normal actions of the economy and therefore it becomes the duty of the physician to discourage the habitual use of alcoholic drinks in all persons of ordinary health, or even in those laboring under occasional depressions, and finally, that great care must be used in discriminating those cases of disease in which it is likely to prove useful.

The book is now put forth in a cheap and popular form, containing marginal notes, which explain the technical terms used in the text. We have no doubt, that among thinking men, it will prove the most powerful coadjutor to the cause of temperance that has ever been given to the public.

*On the Etiology, Pathology and Treatment of Fibro-Bronchitis and Rheumatic Pneumonia.* By Thomas H. Buckler, M. D. Philadelphia. Blanchard & Lea.

Dr. Buckler is well known as an acute and original thinker, a close and accurate observer, and the volume before us is not calculated to detract from his well-earned reputation.

The Doctor's object, in this little book, is to establish the fact, that rheumatic inflammation may and does attack the lungs, as well as any other organ in which fibrous tissue exists. The possibility and extreme probability of such an event seem almost too palpable to demand proof. It is difficult to conceive why a disease, which invades every other fibrous membrane in the body, should spare that only which enters into the composition of the organs of respiration.

The author cites a number of cases, and enters into a critical analysis of them. They are striking and peculiar in their phenomena, and respond in a very remarkable manner to therapeutic agents.

The subject is one of very great importance, and should direct the attention of the profession to this book, which is the only one that gives special heed to it. The author has certainly made out a very strong case.

---

*Lectures on Surgical Pathology, delivered at the Royal College of Surgeons of England.* By James Paget, F. R. S., lately Professor of Anatomy and Surgery to the College, Assistant Surgeon and Lecturer on Physiology at St. Bartholomew's Hospital. Hypertrophy, Atrophy, Repair, Inflammation, Mortification, Specific Diseases and Tumors. Philadelphia. Lindsay & Blakiston, 1853.

Such is the title of one of the most complete treatises on the subject of surgical pathology that has ever issued from the press. Many of these lectures were printed shortly after their delivery, and had a wide circulation. Those on ***reproduction and repair after injuries,*** were published in Rankin's Half Yearly Abstract, had a wide circulation, were much read and greatly admired.

These lectures were founded upon the pathological specimens in the museum of the Royal College of Surgeons, in London, which are designed chiefly to illustrate the principles of general surgical pathology. This volume is not a mere republication of the lectures as delivered. They have been carefully revised and many important additions have been made. The lectures on cancer especially are much fuller than they formerly were, and embody the latest information in regard to this important subject.

It is hardly necessary for us to say any thing of the manner in which these subjects are handled. Mr. Paget's merits are so well known to the reading portion of the medical profession as to make that almost a work of supererogation on our part. He is a well-informed physiologist, and he very judiciously brings the science of the healthy body to bear upon the study of the diseased organism. His style is clear, easy and natural, never disgusting you by vulgarity on the one hand or stilted rhetoric on the other.

The work begins with a consideration of the all-important subject of nutrition, its relations of development and growth, and the conditions of the perfect performance of the function. A foundation having thus been laid in physiology, the pathology of nutrition follows naturally. The disorders of nutrition, excessive on the one hand, resulting in hypertrophy, insufficient on the other, leading to atrophy are then taken up. As a corollary to the latter we have the process of degeneration, especially the atheromatous or fatty degeneration, which has risen to such importance in modern pathology. Then follow the lectures on repair and reproduction, full of important information, exhibiting the natural and healthy reaction of the system against injury. From this the transition is easy to that reaction which passes beyond the limits of health; inflammation, with its products, the disposition the system makes of those products, and its terminations. Specific diseases are then taken up and the discussion of tumors, their classification and history, occupies nine lectures. Cancer is then carefully studied, five lectures, extending over 140 pages, being devoted to its examination. A lecture on tubercles finishes the book.

We cannot close our notice of this very valuable work, without expressing our admiration of the exceedingly handsome manner in which it has been brought out. Like most books issued by Messrs. Lindsay & Blakiston, its typographical execution is faultless. The wood-cuts are unexceptionable. It would be difficult, if not impossible, to excel them even in England, where the art of wood-engraving has been brought to so high a state of perfection.

*A Treatise on the Venereal Disease.* By John Hunter, F. R. S., with copious additions, by Dr. Philip Ricord, Surgeon of the Hospital du Midi, Paris, &c., edited, with notes, by Freeman J. Bumstead, M. D., Physician to the North Western Dispensary, New York. Philadelphia. Blanchard & Lea.

The first glance at this edition of John Hunter's famous treatise, reminds us very forcibly of those pieces of literary patchwork, the pests of our youth, the ***variorum*** editions of the classics, with innumerable and interminable annotations, a little rivulet of text meandering through a continent of notes. The notes too were such multiform things. One ***eruditissimus*** differs so very learnedly and lengthily from another ***vir ornatissi-***

*mus* in regard to the quantity of vowels, and there is such a whirlwind of learned dust, and such hosts of *illustrissimi* are called in to bolster up a particular reading, and such a jangle of *optime Jonesius* and *pessimè Smithius* that you forget the subject of discussion in the warmth and prolixity of the debate, and lose sight of the author in the crowd of his annotations. The present edition was edited in France by Ricord, with numerous additions, and is now re-edited in this favored land by Dr. Bumstead. Not content, however, to re-edit after Ricord, the American goes back to two other English editions.

Shortly after Hunter's death, Sir Everard Home brought out an edition of the great physiologist's work on the venereal disease, which he embellished with annotations of his own and alterations which he professed to have derived from Hunter's manuscripts. In 1835, Mr. George C. Babington issued an edition of the second edition, published during Hunter's lifetime and under his own supervision, adding however most of Home's annotations. Dr. Bumstead uses this edition and copies its notes. Thus the attentive reader, who peruses this edition, finds himself in a wilderness of annotations, marked RICORD, ED. G. C. B., and HOME. To add to the confusion, many of the comments and alterations are introduced into the text, and then these names and initials are placed before them.

Our objections, however, are limited entirely to the *form* of the book. The contents are very valuable. They embrace the experience of several eminent men who have seen much of the disease in question. Ricord is, as is well known to our readers, generally regarded as the highest living authority in all matters pertaining to the recognition, history and treatment of this formidable and disgusting class of disorders. The other gentlemen, from their position, must necessarily have seen much of these affections, so that the reader of this edition has the advantage of the results of the observation of an unusually large number of cases.

*Elements of Chemistry, for the Use of Colleges, Academies and Schools* By M. N. REGNAULT, translated from the French by T. FORREST BETTON, M. D., M. A. N. S., and edited with notes by JAMES C. BOOTH and WILLIAM L. FABER. Second edition, to which is appended a comparative table of French and English Measures. In two volumes. Philadelphia. Clark & Hesser, 1853.

WORKS on Chemistry have multiplied very rapidly within the last three or four years, but the demand for them has by no means diminished. This may be accounted for in two ways. The number of students of chemistry is constantly increasing, and the science itself is rapidly advancing. The text-book which was thought a few years since to be perfectly unexceptionable in the mode of its arrangement is now found to be entirely insufficient. The science has not only advanced, but the relations of its parts have in some measure changed. This state of things may be lamented by those who do not look beneath the surface, but they might as well complain that the boy romping about the house cannot wear clothes of the same size and pattern which suited him perfectly while he was dandled upon his nurse's knee.

There has been, also, it must be confessed, a defect in most of the text-books on this important science, which very much impaired their usefulness to a large class of readers. The connection of the great results of chemistry, with the every-day duties of the manufacturer was not sufficiently elucidated. It was, as a general thing, merely glanced at by way of illustration, or rather carelessly commented on. It constituted no part of the special study of the author, but was only alluded to as a collateral fact. It is true there were works which were devoted especially to technological chemistry, but they were bulky and too prolix for any thing but books of reference. Besides this, they paid too little attention to chemical principles, so that here again, this divorce of principle from practice was kept up. A manual was needed by the general chemical student, which should supply him with both the principles of the science and the application of them to the different purposes of the arts.

This desideratum is amply supplied by the work before us. A part of the description of every substance used in the arts, consists of an account of the manner in which it is made on the large scale, illustrated by excellent sketches of the apparatus used in the manufacture. Nor is it a mere dry, barren, technological description of processes. Much care is taken to impress upon the mind of the reader the *principles* upon which the processes are based. In some instances special experiments are contrived for the express purpose of elucidating these processes.

This renders the work invaluable to that numerous class of readers who are engaged in manufactures into which chemistry enters. This method of treating the subject not only furnishes them with a history of the processes adopted elsewhere, but by expounding the chemical changes taking place at the different stages of these operations, it enables the intellgent manufacturer to improve upon these processes. In this technological department, the additions of the American editors are valuable.

The analytical department of the work is also much fuller than is customary in a treatise on formal chemistry. Processes are given for analysing nearly every compound substance described in the book, and figures of the necessary apparatus are appended.

The author has very judiciously given space for the fuller elucidation of purely chemical actions by throwing aside entirely the chemistry of the imponderables, which occupies so much space that might be better filled in many of our text-books. The truth is, the study of these subjects belongs rather to physics than to pure chemistry, and they have become so extensive as to demand special treatises for their explanation.

Another peculiarity to which we would call special attention is the very large number of engravings (nearly 700) with which this edition is illustrated. They supply most valuable references for forms of apparatus. Nearly every modification of apparatus which the chemist requires, most manufacturing arrangements, and nearly every experiment which can in this manner be exhibited, is figured. The great value of such illustrations it is unnecessary to dwell upon.

After what we have already said, it can scarcely be necessary to add that we most heartily commend this work to all students of chemistry. It is one of the best that has ever come under our observation.

*On the Treatment of Vesico-Vaginal Fistula.* By J. MARION SIMS, M. D. Philadelphia. Blanchard & Lea.

IF ever there was an *opprobrium chirurgicorum,* it has been the disease treated of in the pamphlet before us. Surgeons generally had come at last to the conclusion to let it alone, and hand over its unfortunate victim to the misery and self-abhorrence inseparable from her disgusting complaint.

The author of this little pamphlet, however, has made the relief of these unhappy sufferers the subject of patient, earnest, judicious and successful study. Every thing that is worth any thing in the operation now performed by him is his own. All that had been done before consisted in the only occasionally successful application of the ordinary *armamentarium chirurgicum* to these particular cases. The edges of the wound had been pared and then approximated by sutures and a catheter had been introduced to keep urine from dribbling over the raw surface. There is nothing in all this of any special value.

Dr. Sims has, however, by "patient perseverance in well doing" succeeded in remodelling the operation, or rather in originating a new process. In the first place, by a very simple yet complete arrangement, he exposes the vagina to a clear strong light without inflicting pain on the patient. Secondly, he has contrived a suture which will not cut out and which retains the parts in perfect apposition. Thirdly, he has invented a self-retaining catheter, which is comfortable to the patient and perfectly excludes the urine from the fistula. That is to say, he has overcome all the difficulties and gained much experience in the management of all the details of the case, both before and after the operation.

Of the pamphlet itself it is not necessary to say much. It has been before the public since January, 1852, in the shape of an article in the American Journal of Medical Sciences, of which this is a reprint. It is a clear but modest statement of the methods by which the author proceeds in the performance of the operation and the more difficult subsequent management of the case.

We are glad to see from a little advertisement attached to the pamphlet, that the author has recovered from a severe illness, which, at one time, bade fair to prove fatal, and that he has opened an infirmary for the treatment of this and kindred diseases, at 79 Madison avenue, New York. Long may he live to reap the fruits of his industry and to bring comfort to the afflicted.

*The American Journal of Science and Arts, for November.* Contents: Biography of Berzelius, by Prof. H. Rose, of Berlin; on an Isothermal Oceanic Chart, illustrating the Geographical Distribution of Marine Animals, by James D. Dana; the Coalfield of Bristol County and of Rhode Island, by President E. Hitchcock; Researches on different applications of Magnetic Attraction, by M. J. Nicklés; on the Passivity of Nickel and Cobalt, by M. J. Nicklés; Method of taking Daguerreotype Pictures for the Stereoscope, simultaneously, upon the same plate, with an ordinary camera, by Prof. F. A. P. Bernard; Theoretic Determination of the Expenditure of Heat in the Hot Air Engine, (supplementary article,) by Prof. Frederick A. P. Barnard; on the Consolidation of Coral Formations, by James D. Dana; Re-examination of American Minerals, by J. Lawrence Smith and George J. Brush, Ph. B.; Various actions of Nitric and Oxalic Acids on Salts of Potassium and Sodium, and on Zinc, by J. Lawrence Smith; on the Blood-Corpuscle-holding Cells, and their relation to the Spleen, by W. J. Burnett, M. D.; Extraordinary Fishes from California, constituting a new family, described by L. Agassiz; on a change of Ocean Temperature that would attend a change in the level of the African and South American Continents, by James D. Dana; Reviews and Records in Anatomy and Physiology, by Waldo G. Burnett; Correspondence of M. J. Nicklés; Scientific Intelligence.

In the latter article we find an account of the application of the vapors of ether and chloroform to the steam engine. The experiments were tried in France, and resulted in a very large saving of fuel. The principle consists in making the exhaust steam vaporize the lighter liquid, which vapor is used in the cylinder, thus saving the heat otherwise lost in the transition of steam to water. The saving of fuel amounted to fully 75 per cent. This is a very remarkable result and one which no doubt will encourage further experiment.

Prof. Agassiz's account of the California fishes, like every thing that comes from his pen, is extremely interesting and instructive. Their mode of reproduction is very remarkable, their ***gestation being ovarian,*** the first instance we believe of this sort of gestation in the ***normal*** condition.

*Ellis' Medical Formulary.* Tenth edition, revised, and much extended by Robert P. Thomas, M. D., Professor of Materia Medica in the Philadelphia College of Pharmacy. Philadelphia. Blanchard & Lea, 1854.

Ellis' Formulary has been so long before the profession that it would be a work of supererogation to comment upon it. Every where it has been recognized as a very valuable addition to the library of the physician and of the pharmaceutist.

The present edition is in many respects an improvement upon its predecessors. Complicated formulæ have been simplified. New prescriptions have been added, especially such as contain the new remedies. The formulæ for external application have a book to themselves. The tables of dose, have not only been revised but rewritten. And lastly, all obscurity has been removed by the revision of the old prescriptions, and the alteration of their nomenclature to correspond with that adopted in the United States Pharmacopœia.

The book, as it stands, is one that should lie on every physician's office-table and every druggist's counter. It contains more useful, available, practical information than any work of the size of which we have any knowledge.

*Handbook of Chemistry, Theoretical, Practical and Technical.* By F. A. Abel, Professor of Chemistry at the Royal Military Academy, Woolwich, and Assistant Teacher of Chemistry at St. Bartholomew's Hospital; and C. L. Bloxam, formerly first Assistant to the Royal College of Chemistry. London. John Churchill, Prince's street, Soho, 1854.

This book is constructed upon a plan somewhat new. The first part is devoted to a consideration of those portions of physics which must be understood by the practical chemist. Then comes a very satisfactory description of the various chemical manipulations, sufficient to constitute a safe guide to the student who desires to acquaint himself with the various manual operations of the laboratory. Elementary chemistry is next taken up, and treated concisely but clearly, and lastly the authors give an excellent synopsis of analytical chemistry.

The account of the chemical manipulations is quite full, and technology has received a due share of attention. Processes are given for the convenient and rapid analysis of the chemicals produced on a large scale. The plan adopted, in the sections on analysis, of giving examples for practice, is very well, as far as it goes, but should, we think, have been preceded by a fuller account of general analysis.

However, as the book stands, it will be found a very valuable manual for the chemical student.

***Second Report on Quarantine, Yellow Fever, with Appendices. Presented to both Houses of Parliament by command of her Majesty.*** London: Printed by W. Clowes & Son, for her Majesty's Stationary Office, 1852.

This is an elaborate report upon yellow fever, in which the epidemics at Gibraltar, Boa Vista, Martinique, Cadiz, &c., are examined with much care and attention. The authors of the report come to the conclusion that there is no ground for the belief in the contagious character of this scourge of the tropics, and that the evidence of its importation is, in all cases, entirely insufficient.

In this conclusion they are at issue with the British and Foreign Medico-Chirurgical Review. In the number for October, 1852, there is an able review of this very report, in which directly opposite conclusions are arrived at, from the consideration of the same epidemics.

***Introductory Lecture to the Course for* 1853 *and '4, in the Medical Institution of Yale College.*** By Jonathan Knight, M. D.

This introductory is chiefly taken up with a sketch of the history of the medical school attached to Yale College. It states the very remarkable fact, to which we know no parallel, that out of the *four* professors originally appointed *forty years ago,* three are still living and retain their professorships.

Another peculiarity about this school, which we should be very happy to see other medical colleges adopt, is the independence of the board of examiners of the fees and their influence. At the time the college was chartered, the State society was unwilling to part with the privilege it had hitherto exclusively enjoyed of confirming degrees upon all candidates for a diploma. A compromise was accordingly agreed upon by which the faculty yielded the casting vote in any case to the society, the examining board being composed of both organizations. The benefits of this arrangement, especially the greater security to the community from the murderous blunders of unqualified practitioners, are too manifest to be more than alluded to.

Such a feature in the constitution will do much to secure a higher and more honorable state of feeling in the professors of medical schools. The contemptible huckstering for students which so disgusts every man of tolerable decency, the deliberate violation of charter-provisions will be avoided. A set of professors, acting under the direct supervision of a board of respectable medical men, unconnected with the college and uninfluenced by the paltry consideration of the fees, would hardly dare to be guilty of the shameful act of selling the tickets for a back course of lectures which a student never attended, in order to put a young man, who has not complied with the requisitions of the charter, on a par with those who have; to admit to examination persons who, they know perfectly well, according to their own standards, are unqualified for graduation; thus violating their own laws, flying in the face of the regulations of the National Medical Convention, and deliberately lowering the already depressed standard of requirements, for the petty bribe of fifteen dollars in hand paid to each professor. In spite of any virtuous indignation which may be gotten up in consequence of such an insinuation, we must persist in believing that

—"Mankind are unco' weak
And little to be trusted
If self the wavering balance shake,
'Tis rarely right adjusted;"

especially since we *know* that this thing has been done, and *is* done, and *will* be done, till the watchfulness of the profession prevents it, by schools, the alumni and professors of which would be terribly shocked, should any profane wretch express a doubt of their respectability. We can, if necessary, cite date, place and witnesses for what we assert.

*Principles of Organic and Physiological Chemistry.* By Dr. Carl Löwig, Doctor of Medicine and Philosophy, Ordinary Professor of Chemistry in the University of Zurich, Author of Chemie der Organischen Verbindungen. Translated by Daniel Breed, M. D., of the U. S. Patent Office, late of the Laboratories of Liebig and Löwig. Philadelphia, A. Hart, *late* Carey & Hart.

This book, having been reviewed in the original department of this number of the Journal, needs no further notice in this place.

